# Supplementary material for: Content-rich biological network constructed by mining PubMed abstracts
Source: BMC Bioinformatics. 2004 Oct 8;5:147. doi: 10.1186/1471-2105-5-147 (PMC528731; doi:10.1186/1471-2105-5-147)
Supplement: Additional File 2 — The original results of the above study (non-essential files are deleted to keep the file size under the limit set by BMC bioinformatics). [file 1471-2105-5-147-S2.bz2 › chilibotAdditionalFile2/dip20/231ID7931156E750/html/UL52_UL8.html]

 


 **UL52** and **UL8** 
  
Found 43 abstracts in PubMed, retrieved 20.  
 

 What does Google say? 
 PDF only 
| .edu only 

---

**Interactive relationship** (e.g. stimulation, inhibition, etc)

**Neutral relationship**- Two hybrid analysis of the interaction between the  **UL52**  and  **UL8**  subunits of the herpes simplex virus type 1 helicase primase.  Ref: 10501495 J Gen Virol, 1999
- In contrast, the  **UL52**   **UL8**  interaction gave a strong signal in the two hybrid system.  Ref: 10501495 J Gen Virol, 1999
- The interactions of the  **UL52**  protein with the  **UL8**  and UL5 proteins were analysed by using the yeast two hybrid system.  Ref: 10501495 J Gen Virol, 1999
- Inhibition of herpes simplex virus replication by a 2 amino thiazole via interactions with the helicase component of the UL5  **UL8**   **UL52**  complex.  Ref: 9696789 J Virol, 1998
- The herpes simplex virus type 1  **UL8**  protein influences the intracellular localization of the  **UL52**  but not the ICP8 or POL replication proteins in virus infected cells.  Ref: 8811024 J Gen Virol, 1996
- Deletion analysis showed that a 548 amino acid fragment of  **UL52**  amino acids 366 914 retains the ability to interact with  **UL8** .  Ref: 10501495 J Gen Virol, 1999
- Deletion mutants of the herpes simplex virus type 1  **UL8**  protein effect on DNA synthesis and ability to interact with and influence the intracellular localization of the UL5 and  **UL52**  proteins.  Ref: 9344911 Virology, 1997

**Non-interactive relationship** (e.g. studied together, co-existance, homology, etc.)

- Herpes simplex virus type 1 expresses a heterotrimeric helicase primase, the subunits of which are encoded by the viral UL5,  **UL8**  and  **UL52**  genes.  Ref: 10501495 J Gen Virol, 1999
- The DNA helicase primase complex of HSV 1 consists of three sub units with molecular weights of 1 0 00 and 7 00, encoded by genes  **UL52** , UL5 and  **UL8** , respectively.  Ref: 9421876 Virus Genes, 1997
- Herpes simplex virus type 1 encodes a helicase primase complex composed of the products of the UL5,  **UL52** , and  **UL8**  genes.  Ref: 8662872 J Biol Chem, 1996
- Since this pattern can form in cells infected with mutants which are defective in UL5,  **UL8** , UL9, or  **UL52**  in the presence of polymerase inhibitors C.  Ref: 9151871 J Virol, 1997
- The primase activity of the intertypic complex was 10 fold lower than that of HSV 1 UL5 HSV 1  **UL8**  HSV 1  **UL52** .  Ref: 9445019 J Virol, 1998
- Herpes simplex virus type 1 encodes a heterotrimeric helicase primase composed of the products of the UL5,  **UL52** , and  **UL8**  genes.  Ref: 9020191 J Biol Chem, 1997
- All three subunits of this complex UL5,  **UL8** , and  **UL52**  are required for viral DNA replication in all cell types.  Ref: 9445019 J Virol, 1998
- We show that the HSV 1 UL5,  **UL8** , UL29, UL30, UL42, and  **UL52**  gene products along with the AAV Rep68 protein are sufficient to initiate replication on duplex DNA containing the AAV origins of replication, resulting in products several hundred nucleotides in length.  Ref: 11581393 J Virol, 2001
- Herpes simplex virus type 1 encodes a heterotrimeric helicase primase complex composed of the products of the UL5,  **UL52** , and  **UL8**  genes.  Ref: 11278618 J Biol Chem, 2001
- The herpes simplex virus type 1 DNA helicase primase is a heterotrimer encoded by the UL5,  **UL8** , and  **UL52**  genes.  Ref: 8702953 J Biol Chem, 1996
- The intertypic complex HSV 2 UL5 HSV 1  **UL8**  HSV 1  **UL52**  was purified and biochemically characterized.  Ref: 9445019 J Virol, 1998
- The UL5,  **UL8** , and  **UL52**  genes of herpes simplex virus type 1 encode a multisubunit assembly that possesses primase, DNA helicase, and DNA dependent nucleoside triphosphatase activities.  Ref: 9013584 J Biol Chem, 1997
- T157602 inhibited reversibly the helicase activity of the HSV UL5  **UL8**   **UL52**  UL5 8 52 helicase primase complex with an IC50 concentration of compound that yields 50% inhibition of 5 microM.  Ref: 9696789 J Virol, 1998
- While the  **UL8**  component of the helicase primase is not required for enzymatic activities by a subassembly of the UL5 and  **UL52**  proteins, only the holoenzyme UL5 8 52 was stimulated by ICP8.  Ref: 9129659 J Gen Virol, 1997
- The results shown in this paper are consistent with the model that the five proteins, ICP8, UL5,  **UL8** , UL9, and  **UL52** , form a scaffold.  Ref: 12634381 J Virol, 2003
- The ordered assembly of the herpes simplex virus HSV type 1 replication apparatus leading to replication compartments likely involves the initial assembly of five viral replication proteins, ICP8, UL9, and the heterotrimeric helicase primase complex UL5  **UL8**   **UL52** , into replication foci.  Ref: 12634381 J Virol, 2003
- The herpes simplex virus type 1 UL5,  **UL8** , and  **UL52**  genes encode an essential heterotrimeric DNA helicase primase that is responsible for concomitant DNA unwinding and primer synthesis at the viral DNA replication fork.  Ref: 9593724 J Biol Chem, 1998
- The assembly of complete HSV replication compartments and incorporation of BrdU were both abolished by treatment with phosphonoacetic acid and by omission of any one of the seven viral replication proteins, UL5,  **UL8** , UL9, UL42,  **UL52** , SSB, and Pol, that are essential for viral DNA replication.  Ref: 9060678 J Virol, 1997
- The herpes simplex virus type 1 HSV 1 helicase primase, an essential component of the viral DNA replication machinery, is a trimeric complex of the virus coded UL5,  **UL8** , and  **UL52**  proteins.  Ref: 9344911 Virology, 1997
- An assembly of the UL5 and  **UL52**  subunits retains both enzymic activities, and the  **UL8**  protein has been implicated in modulating these functions, facilitating efficient nuclear uptake of the complex and interacting with other viral DNA replication proteins.  Ref: 9344911 Virology, 1997
- The ability of the  **UL8**  mutants to facilitate efficient nuclear localization of  **UL52**  in the presence of coexpressed UL5 was examined by immunofluorescence.  Ref: 9344911 Virology, 1997
- In BHK cells infected with ambUL8, a mutant with an amber termination codon within the coding region of gene  **UL8** , the  **UL52**  protein did not enter the nucleus, although ICP8 and POL entered the nucleus in a normal fashion.  Ref: 8811024 J Gen Virol, 1996
- Herpes simplex virus type 1 encodes a heterotrimeric helicase primase complex that is composed of the products of the UL5,  **UL52** , and  **UL8**  genes.  Ref: 10075707 J Biol Chem, 1999
- The Herpes simplex virus type 1 primosome consists of three subunits that are the products of the UL5,  **UL8** , and  **UL52**  genes.  Ref: 9278436 J Biol Chem, 1997
- the heterotrimeric helicase primase complex, encoded by UL5,  **UL8** , and  **UL52**  genes, is believed to unwind duplex viral DNA at replication forks.  Ref: 11642367 Prog Nucleic Acid Res Mol Biol, 2001
